# Amyloid-Like Aggregates of the Yeast Prion Protein Ure2 Enter Vertebrate Cells by Specific Endocytotic Pathways and Induce Apoptosis

**DOI:** 10.1371/journal.pone.0012529

**Published:** 2010-09-02

**Authors:** Chen Zhang, Antony P. Jackson, Zai-Rong Zhang, Yan Han, Shun Yu, Rong-Qiao He, Sarah Perrett

**Affiliations:** 1 National Laboratory of Biomacromolecules, Institute of Biophysics, Chinese Academy of Sciences, Beijing, China; 2 Department of Biochemistry, University of Cambridge, Cambridge, United Kingdom; 3 Department of Neurobiology and the Sino-Japan Joint Laboratory of Neurodegenerative Diseases, Beijing Institute of Geriatrics, Xuanwu Hospital of the Capital University of Medical Sciences, Beijing, China; 4 State Key Laboratory of Brain and Cognitive Science, Institute of Biophysics, Chinese Academy of Sciences, Beijing, China; 5 Graduate University of the Chinese Academy of Sciences, Beijing, China; Ohio State University, United States of America

## Abstract

**Background:**

A number of amyloid diseases involve deposition of extracellular protein aggregates, which are implicated in mechanisms of cell damage and death. However, the mechanisms involved remain poorly understood.

**Methodology/Principal Findings:**

Here we use the yeast prion protein Ure2 as a generic model to investigate how amyloid-like protein aggregates can enter mammalian cells and convey cytotoxicity. The effect of three different states of Ure2 protein (native dimer, protofibrils and mature fibrils) was tested on four mammalian cell lines (SH-SY5Y, MES23.5, HEK-293 and HeLa) when added extracellularly to the medium. Immunofluorescence using a polyclonal antibody against Ure2 showed that all three protein states could enter the four cell lines. In each case, protofibrils significantly inhibited the growth of the cells in a dose-dependent manner, fibrils showed less toxicity than protofibrils, while the native state had no effect on cell growth. This suggests that the structural differences between the three protein states lead to their different effects upon cells. Protofibrils of Ure2 increased membrane conductivity, altered calcium homeostasis, and ultimately induced apoptosis. The use of standard inhibitors suggested uptake into mammalian cells might occur via receptor-mediated endocytosis. In order to investigate this further, we used the chicken DT40 B cell line DKOR, which allows conditional expression of clathrin. Uptake into the DKOR cell-line was reduced when clathrin expression was repressed suggesting similarities between the mechanism of PrP uptake and the mechanism observed here for Ure2.

**Conclusions/Significance:**

The results provide insight into the mechanisms by which amyloid aggregates may cause pathological effects in prion and amyloid diseases.

## Introduction

Amyloid diseases are characterized by misfolding and aggregation of normal soluble peptide into β-sheet-rich oligomeric structures and amyloid fibrils in specific tissues [1]. Among amyloid diseases, prion diseases are unique in that the pathology can be transmitted by an infectious process involving misfolding of the prion protein to form an infectious state [2], [3]. Prions are infectious proteins that can transmit biological information by propagating protein misfolding and aggregation. However, the underlying pathological mechanism remains poorly understood.

Proteins that are not associated with disease can in some cases be induced under extreme conditions to transform into amyloid-like aggregates; the cytotoxic properties of the amyloid-like aggregates formed from non-disease-related proteins [4] are closely similar to those of disease-associated proteins such as the Aβ peptides, α-synuclein, and transthyretin [5]–[9]. This then suggests that the cytotoxic properties may be determined by common structural features of certain types of aggregates, rather than by specific amino acid sequences. Increasing evidence suggests that protofibrils, which are intermediates in the amyloid fibril formation process, rather than mature fibrils, constitute the toxic species [10], [11].

Common histological findings include formation of aberrantly folded proteins into extracellular plaques or intracellular inclusions [12]. A deeper understanding of the detailed mechanism of protein aggregation and the resulting cellular toxicity should lead to rational drug design for this type of disease. Protein aggregates, in the form of amyloid plaques, neurofibrillary tangles, and/or intracytoplasmic or intranuclear inclusions, are thought to lead to damage of the cell membrane and disruption of ion homeostasis, induction of apoptosis, and finally, cell death [13].

Ure2 is the protein determinant of the epigenetic factor [*URE3*] of *Saccharomyces cerevisiae*, which has been demonstrated to represent a prion of yeast [14], [15]. Analogous to the mammalian prion protein [2], Ure2 is aggregated and protease-resistant in prion strains [14], [15]. However, unlike mammalian prions, which exhibit cytotoxicity in their aggregated form, Ure2 aggregates do not show any marked toxic effect towards yeast cells. Ure2 forms amyloid-like filaments *in vitro*, which share several morphological, structural, and tinctorial features with amyloids, including enhanced resistance to proteolysis, increased Thioflavin T (ThT) fluorescence, and yellow-green birefringence in cross-polarized light upon Congo red binding [16]–[19]. However, an unusual property of Ure2 fibrils is their ability to maintain native-like structure and activity within the fibrillar arrays [20]–[22].

Ure2 is a 354-amino acid cytoplasmic homodimeric protein consisting of a relatively flexible and protease-sensitive N-terminal region (∼90 amino acids), and a globular C-terminal region [17], [23]–[25]. The N-terminal region is required for its prion properties *in vivo* [15] and to form amyloid-like filaments *in vitro* [16], [26], [27]. The C-terminal region shows structural similarity to glutathione transferases (GSTs) [24], [25], [28], has glutathione-dependent peroxidase activity [20], glutaredoxin activity [22], and is necessary for the regulatory function of Ure2 *in vivo*: Ure2 interacts with the transcription factor Gln3 allowing control of nitrogen catabolite repression, blocking the uptake of poor nitrogen sources in the presence of a good nitrogen source [29], [30].

The easy availability of different conformational states of Ure2 makes it an ideal model to investigate molecular features of the toxicity of protein assemblies. Pieri and colleagues [31], [32] previously examined the effect of Ure2 aggregates on murine H-END cells and found that different states of Ure2 aggregates were toxic regardless of their aggregation state, which differs from what has been proposed for amyloids, where toxicity follows extensive molecular rearrangements and is usually restricted to protofibrillar aggregates.

Here we investigated further the effect of Ure2 aggregates towards a variety of mammalian cells lines, and the mechanism of cytoxicity, in order to differentiate cell-specific effects from the generic properties of particular types of amyloid-like aggregates. In addition, we investigated the uptake pathway using a chicken DT40 B cell line (DKOR), which allows conditional expression of clathrin [33]. The results may shed light on the pathologic mechanism and potential treatment strategies for prion and amyloid diseases.

## Results

### Protofibrils of Ure2 significantly inhibited the growth of four cell lines in a dose-dependent manner

**Figure 1:**
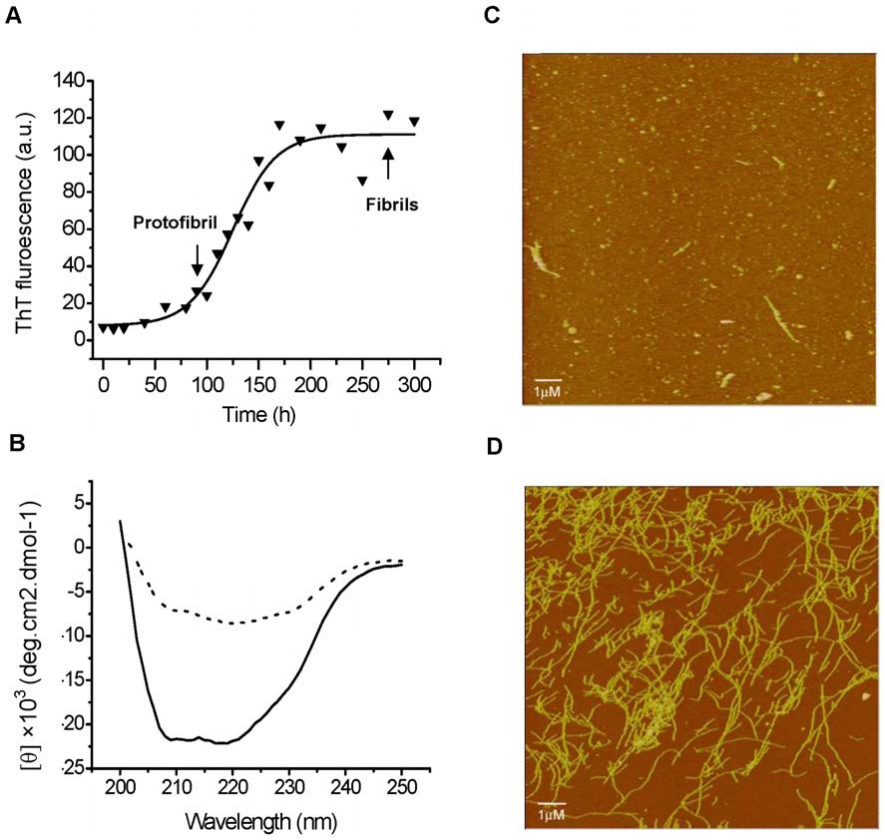
Formation of amyloid-like structure for Ure2. Fibril formation was monitored by binding of the fluorescent dye ThT, AFM and CD. (**A**) Kinetics of formation of amyloid-like structure for Ure2 monitored by ThT binding. (**B**) Far-UV CD spectra of 20 µM native Ure2 and protofibrils in 50 mM Tris-HCl buffer containing 0.2 M NaCl (pH 7.5). 20 µM Native Ure2 (−), 20 µM protofibrils of Ure2 (…). (**C**), (**D**) Morphology of amyloid-like structures monitored by AFM for WT Ure2. (**C**) Protofibrils (height 3–10 nm) were abundant at early exponential growth phase of the fibril growth curve. (**D**) Mature fibrils (height 12–15 nm) were predominant at later time points (plateau phase).

The sigmoidal time course of formation of amyloid-like fibrils of Ure2 is conveniently monitored using the fluorescent dye Thioflavin T (ThT) [18]. Combining ThT binding fluorescence and imaging by atomic force microscopy (AFM), different fibril types of Ure2 (defined by morphology and/or thickness) are observed to appear in a time dependent manner [27]. In this study, fibrils were grown under conditions where fibril growth is relatively slow (incubation at 4°C without agitation) and so appearance of different fibril types is well separated in time (Fig. 1A). Protofibrils were defined as small fibrillar aggregates appearing early during the time course *i.e.* during the exponential growth phase of the fibril growth curve (Fig. 1A) and having thickness (*i.e.* height) of 3–10 nm, as measured by AFM (Fig. 1C). Mature fibrils were defined as the longer, thicker fibrils that are abundant in the final plateau phase of fibril grown (Fig. 1A), with a height of 12–15 nm measured by AFM (Fig. 1D). Far-UV circular dichroism (CD) spectra indicated that in contrast to native Ure2, which shows a typical CD spectrum for an α–helical protein, the protofibrils show a clear decrease in the relative α–helical content (Fig. 1B).

**Figure 2:**
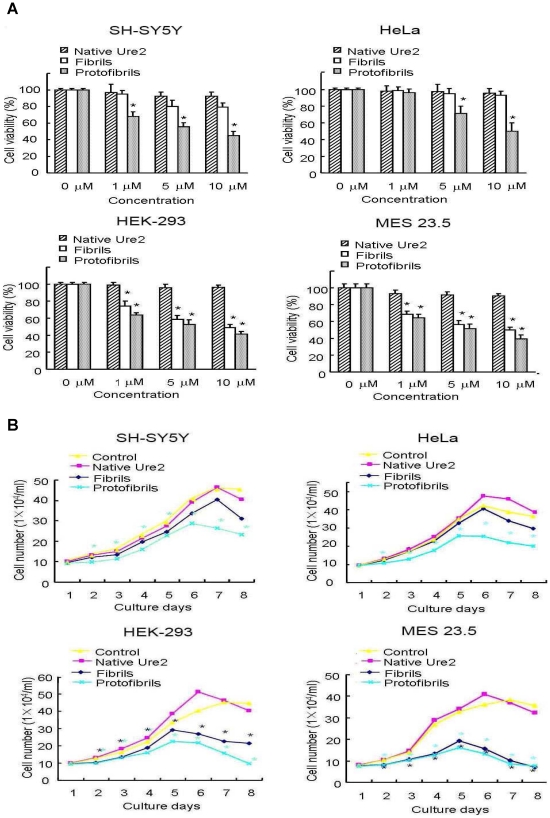
Effects of Ure2 aggregates on the cell viability and proliferation of four cultured cell lines. The cell lines were SH-SY5Y, HEK-293, HeLa and MES 23.5. *P<0.01 compared with the control group. The results shown are the mean of at least three independent measurements and the error bars represent the S.E. of the mean. (**A**) MTT assay of cell viability after incubation for 48 h with different aggregated states of Ure2 protein. The concentration of Ure2 species shown in the x-axis of the figure was determined as described in the Materials and Methods. (**B**) Growth curves of the cell lines in the presence of Ure2 aggregates. The concentrations of Ure2 species were 3 µM.

**Figure 3:**
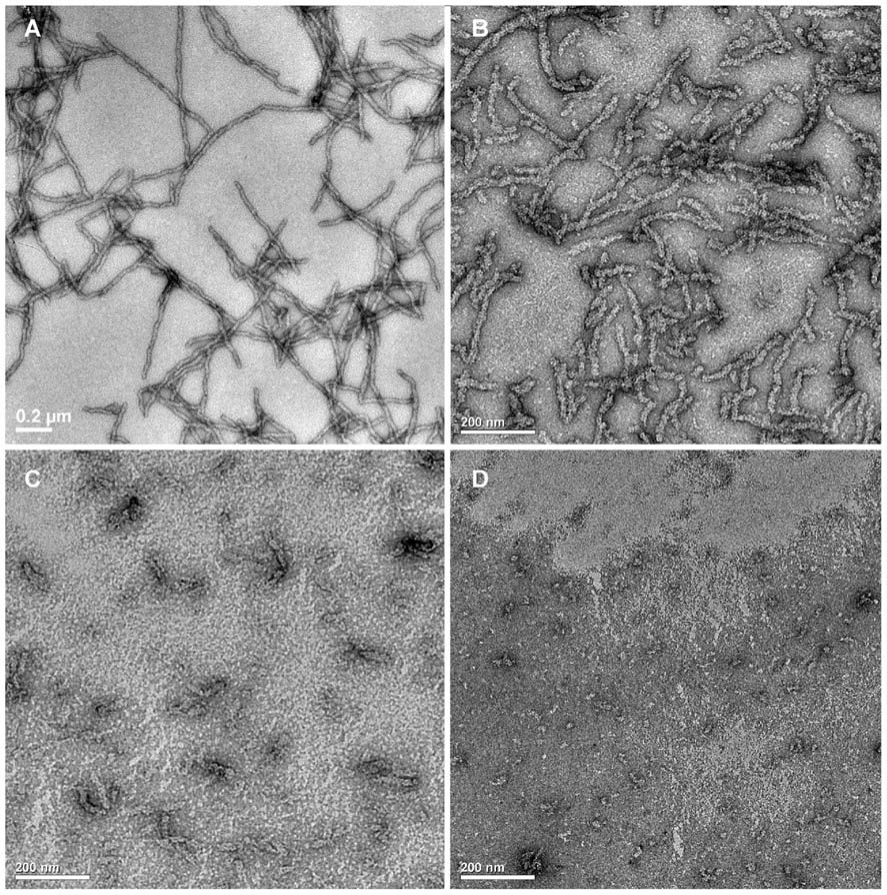
Comparison of the morphology of Ure2 aggregates before and after incubation under the conditions of the cell culture experiments. Electron microscopy images of negative-stained Ure2 fibrils and protofibrils are shown. Scale bars  = 200 nm. (**A**) Freshly prepared mature fibrils of Ure2. (**B**) Mature fibrils of Ure2 after incubation in DMEM for 5 days. (**C**) Freshly prepared protofibrils of Ure2. (**D**) Protofibrils of Ure2 after incubation in DMEM for 5 days.

We chose four cell lines: human SH-SY5Y neuroblastoma cells, rat MES 23.5 dopaminergic neural cells, human HEK-293 embryonic kidney cells and human HeLa adenocarcinoma cells. We then tested the effect of native Ure2 protein, protofibrils and mature fibrils on the viability of the four cell lines, when added to the extracellular medium. MTT assay results showed that addition of protofibrils decreased the viability of all four cell lines in a dose dependent manner (Fig. 2A). An equivalent concentration of mature fibrils had less effect on cell viability than protofibrils in HEK-293 and MES 23.5 cells, and mature fibrils showed no cytotoxic effect in SH-SY5Y and HeLa cells (Fig. 2A). As a control, native Ure2 had no effect on any of the cell lines (Fig. 2A). The decrease in cell viability (Fig. 2A) was found to correlate with a decrease in cell number (Fig. 2B). As a control, no obvious morphological change was observed after incubating mature fibrils or protofibrils under the conditions of the cell culture experiments for 5 days (Fig. 3), indicating that the different fibrillar forms are stable under the conditions of the cell culture experiments. The observation of dose-dependent inhibition of growth of mammalian cells after addition of Ure2 protofibrils, with a significantly lower toxic effect of mature fibrils, agrees with the finding in murine H-END cells [31]. However, a surprising finding in the previous study was that native Ure2 was found to be toxic towards murine H-END cells [31]. The above results suggest that although different cell types showed different sensitivity towards mature fibrils of Ure2, protofibrils of Ure2 consistently induce significant inhibition of mammalian cell growth, in agreement with previous results for a wide variety of disease-related and non-disease related proteins [4]–[9]. These results support a model whereby particular structural characteristics of aggregates formed early in the course of amyloid formation convey toxic properties towards the cells [10], [11].

### Native, protofibrillar, and fibrillar Ure2 can enter mammalian cells

**Figure 4:**
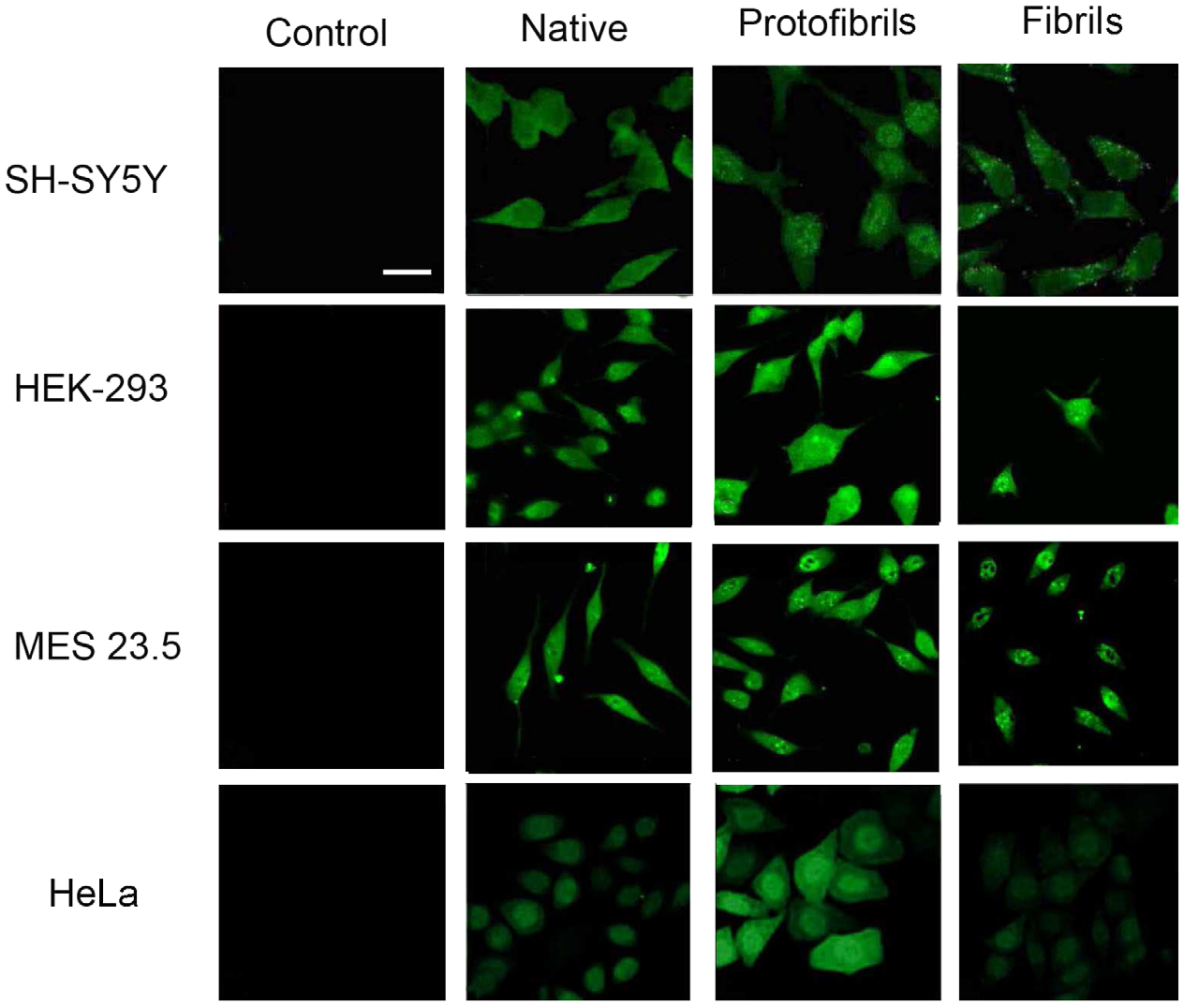
Immunofluorescence labeling of Ure2 after extracellular addition to the cell lines. Labeling with Ure2 polyclonal antibody was carried out in the SH-SY5Y, HEK-293, HeLa and MES 23.5 cell lines 48 h after extracellular addition of different states of Ure2 protein (bar = 12 µm). The concentration of Ure2 and its fibrillar species used here was 3 µM. The images shown are cell sections obtained by confocal microscopy, as described in the Materials and Methods.

Immunofluorescence was carried out to investigate whether the different states of Ure2 could enter into the four mammalian cell lines. Potentially, the localization of the aggregates could shed light on the toxic mechanism, by identifying the organelle where Ure2 conveys its effect. The results demonstrate that the different states of Ure2 could each enter into the four cell lines but to different extents (Fig. 4), with native Ure2 only present diffusely in the cytoplasm, while protofibrils entered into the nucleus and cytoplasm in SH-SY5Y, HeLa and MES 23.5 cells, with observation of immnofluorescent-positive particles in SH-SY5Y and MES 23.5 cells. Particles were also observed in the cytoplasm of SH-SY5Y and MES 23.5 cells in the fibril treated group (Fig. 4).

### Involvement of endocytotic pathways in uptake of Ure2 into mammalian cells

**Figure 5:**
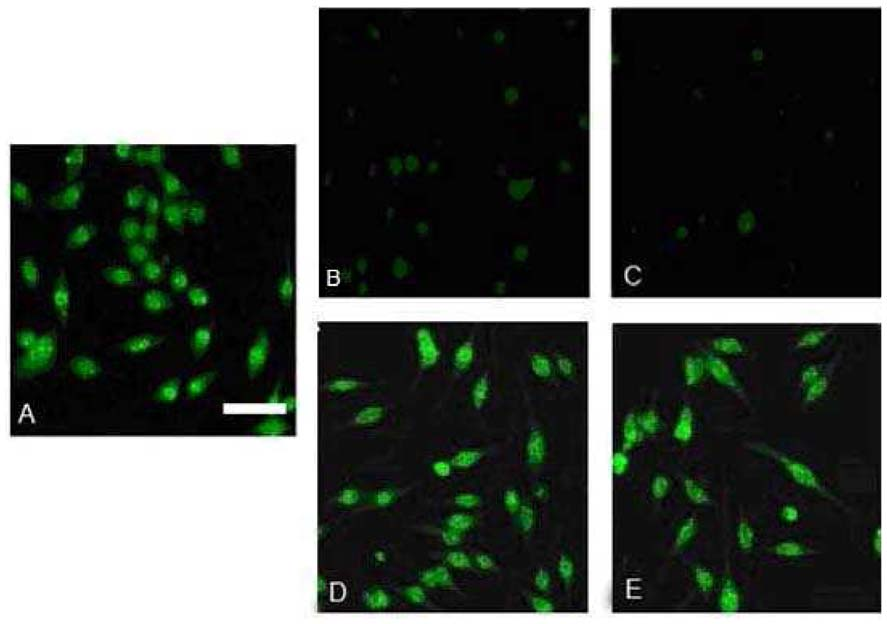
Inhibition of endocytotic uptake of Ure2 protofibrils. 3 µM Ure2 protofibrils and nystatin, filipin, nocodazole or cytochalasin D was added extracellularly to MES 23.5 cells. After 48 h incubation, the cells were subjected to immunofluorescence assay (bar = 25 µm). (**A**) Control (no inhibitor). (**B**) 100 µg/ml nystatin. (**C**) 5 µg/ml filipin. (**D**) 50 µg/ml cytochalasin D. (**E**) 50 µmol/l nocodazole.

Having established that Ure2 could enter cells, in order to investigate the mechanism of uptake of Ure2 further, we used four different inhibitors that are considered to interfere with endocytotic pathways: nystatin (which disrupts lipid raft-mediated endocytotic pathways), filipin (an inhibitor of caveolae-mediated endocytosis), cytochalasin D (an inhibitor of macropinocytosis) and nocodazole (an inhibitor of microtubule-mediated endocytosis) to assay the involvement of endocytotic pathways in the uptake of Ure2 into cells. The results showed that nystatin and filipin could inhibit the uptake of the aggregates into cells (Fig. 5B and C). However, cytochalasin D and nocodazole did not affect uptake (Fig. 5D and E). While use of such inhibitors gives only a crude indication, this nevertheless suggests that Ure2 aggregates may enter cells via specific endocytotic pathways, including lipid raft-mediated and/or caveolae-mediated pathways.

### Role of clathrin in endocytosis of Ure2 aggregates

**Figure 6:**
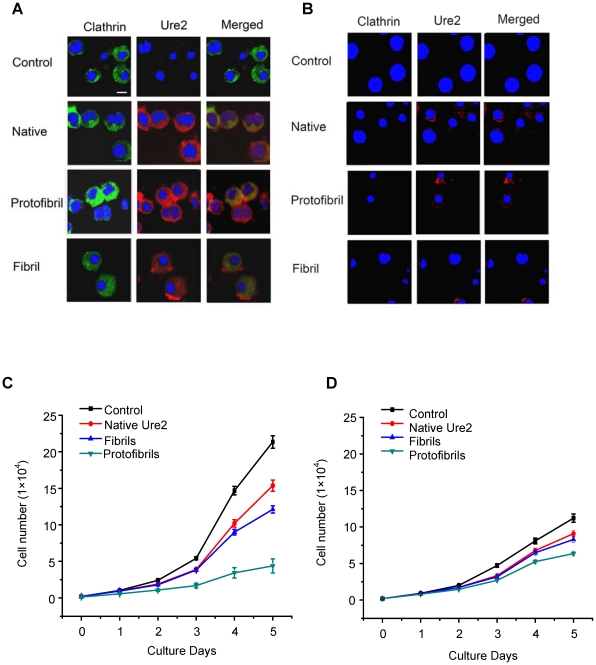
Effects of Ure2 aggregates on the DKOR cell line. (**A**), (**B**) Immunofluorescence co-labeling for clathrin and Ure2 in the DKOR cell line at 48 h after extracellular addition of 3 µM different states of Ure2. Green fluorescence is clathrin, red is Ure2 and blue is Hoechst 33258 nuclear stain (bar = 12 µm). (**A**) DKOR cells with expression of clathrin. (**B**) DKOR cells without expression of clathrin. (**C**), (**D**) Growth curves of the DKOR cell line in the presence of 3 µM Ure2 or its amyloid aggregates. The error bars represent the S.E. of the mean of three independent measurements. (**C**) DKOR cells with expression of clathrin. (**D**) DKOR cells without expression of clathrin.

In order to investigate the uptake pathway of Ure2 aggregates in more detail, we used the DKOR cell line. The DKOR cell line was derived by inactivation of both endogenous alleles of chicken clathrin heavy chain in the DT40 B cell line followed by introduction of human clathrin cDNA under the control of a tetracycline-regulatable expression system (Tet-Off) [33]. Thus using the DKOR cell line, it is possible to assess the role of clathrin in the uptake of specific molecules. Immunofluorescence results using Ure2 polyclonal antibody showed that native Ure2, protofibrils and mature fibrils could all enter into DKOR cells when clathrin was expressed (Fig. 6A). However, in DKOR cells without expression of clathrin, a significant decrease in the extent of uptake of the three states was apparent (Fig. 6B). When the effect of different states of Ure2 on cell growth was examined, protofibrils were found to show the most marked inhibition on growth of DKOR cells, and this was most apparent when clathrin was expressed (Fig. 6C and D).

**Figure 7:**
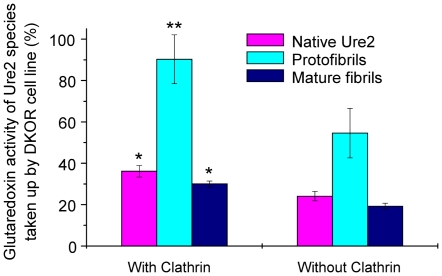
Quantification of the amount of Ure2 entering into the DKOR cell line using enzymatic activity of Ure2. Cells were assayed 48 h after extracellular addition of different states of Ure2. The percentage on the y-axis represents the ratio of glutaredoxin activity of Ure2 species within DKOR cells to the total activity of Ure2 species added into the cell culture. Lysates of clathrin-expressing and clathrin-depleted cells which were not incubated with Ure2 or aggregates were assayed as controls. The glutaredoxin activity of Ure2 or its aggregates in the cell lysates is shown after subtraction of the activity of the control. The reaction conditions are described in the Materials and Methods. The results shown are the mean of three independent measurements and the error bars represent the S.E. of the mean. *P<0.01 compared with protofibril group in DKOR cells with expression of clathrin. **P<0.05 compared with DKOR cells without expression of clathrin.

In order to quantify the extent of uptake, we made use of the enzymatic activity of the Ure2 protein, which is maintained in its fibrillar state [20], [22]. Using the glutaredoxin activity of Ure2 to determine the extent of uptake, we observed that uptake of all three states of the Ure2 protein was significantly reduced in the absence of clathrin (Fig. 7). More striking though, was that the amount of protein entering cells (and detectable after cell lysis and assay of enzymatic activity) was significantly greater for protofibrils than for either mature fibrils or native Ure2; this observation was the same in the presence or absence of clathrin expression.

### Protofibrils increased membrane conductivity in a concentration dependent manner

**Figure 8:**
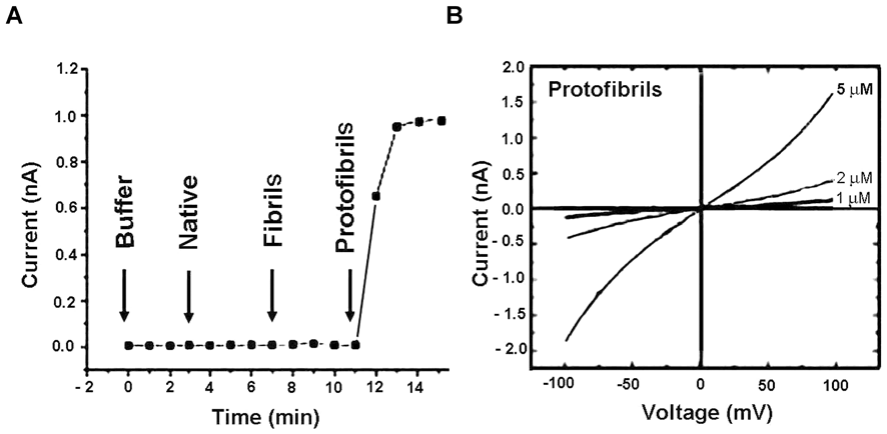
Effect of Ure2 aggregates on membrane conductivity of SH-SY5Y cells. (**A**) Protofibrils induced increase of membrane conductivity. Buffer, 1 µM native Ure2, 1 µM fibrils and 1 µM protofibrils of Ure2 were added sequentially. (**B**) Protofibrils increased the membrane conductivity in a concentration dependent manner. Currents were recorded in response to voltage ramps from −100 to +100 mV at a rate of 50 mV/s. Curves are labeled with the concentration of Ure2 protofibrils used in the assay.

Interaction between amyloid aggregates and the cell membrane has been postulated to play an important role in the neuropathology of Alzheimer's, Parkinson's and prion diseases [34]. We used patch clamp to investigate the effect on the cell membrane of mammalian cells induced by different states of Ure2. The *I-V* curve showed that protofibrils increased membrane conductivity of SH-SY5Y cells, whereas native Ure2 and fibrils had no effect (Fig. 8A). Further, the effect of protofibrils was concentration-dependent (Fig. 8B).

### Protofibrils of Ure2 cause an increase in intracellular free Ca^2+^

**Figure 9:**
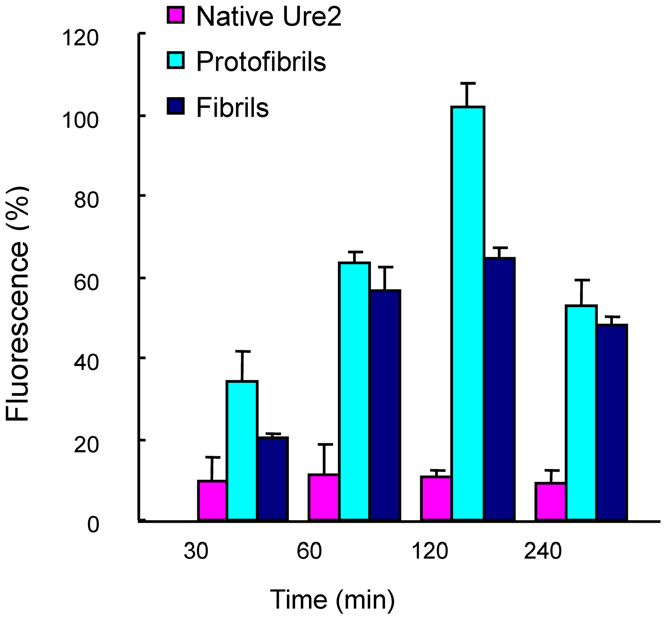
Protofibrils induced a concentration change in intracellular free Ca^2+^ in SH-SY5Y cells. Analysis was performed using Quantity One software (Bio-Rad). The maximum intensity of fluorescence was defined as 100%. The results shown are the mean of at least three independent measurements and the error bars represent the S.E. of the mean.

Alteration of intracellular ion homeostasis is one of the earliest and most common biochemical changes observed in cells exposed to amyloids [35]. To examine the effects of Ure2 aggregates on intracellular free Ca^2+^, we added protofibrils, fibrils or native Ure2 extracellularly to Fluo-3-loaded SH-SY5Y cells. The result showed that protofibrils caused an increase in the intracellular Ca^2+^ from about 30 min after addition, reaching a maximal level at around 2 h, after which the Ca^2+^ level decreased again (Fig. 9). Mature fibrils of Ure2 also caused an increase in intracellular Ca^2+^, with a similar time profile to protofibrils, but the effect was not as large, while native Ure2 evoked no significant change (Fig. 9).

### Protofibrils of Ure2 induced cell apoptosis

**Figure 10:**
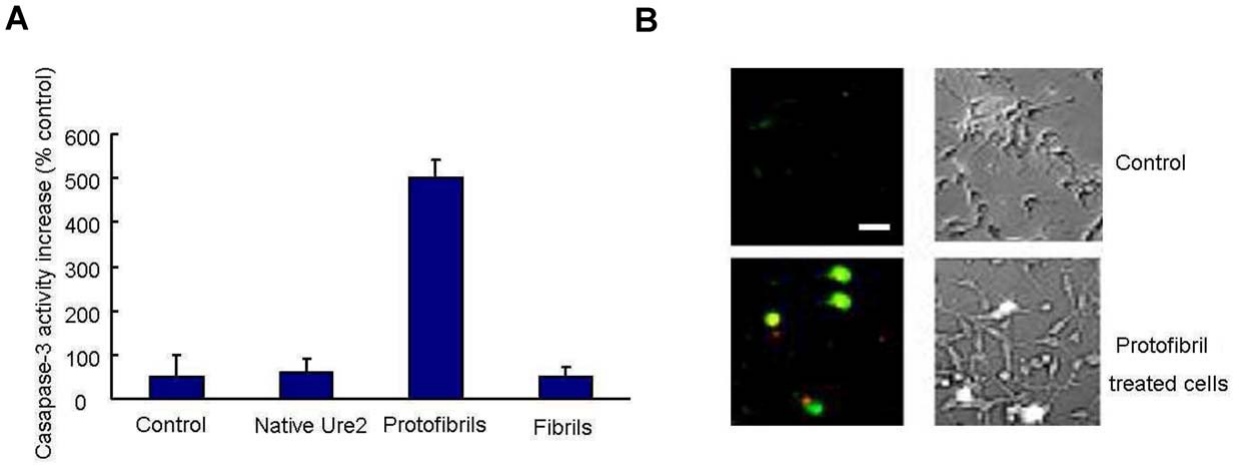
Protofibrils of Ure2 induced apoptosis of SH-SY5Y cells. (**A**) Caspase-3 activity of SH-SY5Y cells increased 5 times with respect to the control group after treatment with protofibrils. The error bars represents the S.E. of the mean of at least three independent measurements. (**B**) Apoptosis assay of SH-SY5Y cells after treatment with protofibrils. Green represents annexin V staining, indicating apoptosis. Red represents propidium iodide staining, indicating necrosis. The cells shown are representative for a total of 10 fields (more than 100 cells) that were imaged (bar = 12 µm).

Apoptosis and necrosis of cells may both be triggered by amyloid aggregate toxicity [36]–[38]. We therefore investigated whether the changes in membrane conductivity and free Ca^2+^ levels induced by Ure2 aggregates lead to cell death, and if so, whether activation of apoptosis was involved. We measured the activity of caspase-3, an important element in signaling transduction leading to apoptosis [39]. After incubation with protofibrils, the caspase 3 activity of SH-SY5Y cells was found to increase 5-fold compared with the control group (Fig. 10A). This is consistent with the effect seen in murine H-END cells [31]. Early apoptotic cells are characterized by exposure of phospholipid normally found in the inner membrane, whereas necrotic cells undergo membrane rupture. We used annexin V and propidium iodide (PI) double labeling to detect phosphatidylserine externalization and membrane integrity in our cell model. Under these conditions, protofibril-treated cells displayed high annexin V and low PI binding, indicating apoptosis rather than necrosis (Fig. 10B). These results indicate that protofibrils of Ure2 cause cell death by triggering the apoptotic pathway, rather than by causing necrosis.

## Discussion

The *S. cerevisiae* prion protein Ure2 forms amyloid-like filaments *in vitro*, which share similar morphological, structural, and tinctorial features with amyloids [16]. In this study, we used Ure2 as a generic model to investigate aspects of the underlying mechanisms of amyloid and prion diseases. A particular advantage of using an exogenous protein such as Ure2 in this type of study, is that the lack of Ure2 present in the original cell-lines means that its uptake can be clearly and unambiguously followed. Another advantage of Ure2 is that the time course of fibril formation is well studied and relatively easily controlled, allowing separation of different types of aggregates for comparative study [27]. In this study, protofibrils were defined as aggregates with diameter 3–10 nm (Fig. 1), similar to those observed for many different types of amyloids by electron and atomic force microscopy [40]–[42]. Mature fibrils were abundant in the final plateau phase of fibril growth and had a height of 12–15 nm (Fig. 1). This pattern of distribution of fibril morphologies has been observed for many amyloidogenic proteins, including islet amyloid [41], α-synuclein [42] and non-disease associated “neoamyloids” [4].

In order to study the effect of different aggregate types on mammalian cells, and to be able to differentiate between general toxic effects of amyloid aggregates, versus effects that might be cell specific, we chose four different cell lines: SH-SY5Y, MES23.5, HEK-293 and HeLa (see Materials section for further details). The results show that the different aggregation states of the Ure2 protein conveyed different effects on mammalian cells: protofibrils significantly inhibited the growth of the cells in a dose-dependent manner, mature fibrils showed less toxicity than protofibrils, while the native state had no effect on cell growth. This suggests that cytotoxicity reflects specific structural characteristics that are present in the protofibril state, consistent with the conclusions drawn from studies using a wide variety of disease-related and non-disease related proteins [4]–[9],[43]. The finding here that native Ure2 is benign towards a range of mammalian cell types, unlike the previously reported effects on murine H-END cells [31], suggests that murine H-END cells may have unique characteristics in their response to the heterologous protein Ure2, that are not representative of neuronal and other cell types. The observation of different susceptibility among different cell lines to the cytotoxic effects of the aggregates may be related to variations in the glycerol phospholipid content of the cell membrane [44], cell differentiation [45], or other cell-specific factors. The lower levels of cholesterol in the membrane of H-END cells may be related to its high susceptibility to Ure2 aggregates. Similarly, the higher level of cholesterol in the membrane of HeLa cells may explain their reduced susceptibility to the toxic effects of Ure2 aggregates compared to the other cell lines examined in this study.

In order to determine whether the cytotoxic effects of Ure2 aggregates were exerted from inside or outside the cells, we followed the fate of the Ure2 protein by immunofluorescence labeling using an antibody that recognizes both native and fibrillar states of Ure2. The results demonstrate that the three different states of Ure2 entered into the four cell lines to different extents (Fig. 4). In general, the accumulation of protofibrils and fibrils within the cells was more apparent than for native Ure2. In the previous study using murine H-END cells, mature fibrils could not enter into cells but only adsorbed on to the plasma membrane, while protofibrils and native dimer were observed both intracellularly and on the membrane [31]. Clearly, protofibrils have an enhanced and general ability to enter cells, whereas the uptake of mature fibrils seems to be more susceptible to cell-specific factors.

The mechanism of uptake into cells is of particular interest, as internalization of PrP is thought to be involved in the mechanism of prion disease [46], [47] and this also has implications for the mechanism of damage caused by extra-cellular amyloid deposits in diseases such as Alzheimer's [48]. We found that regardless of the aggregation state, Ure2 could enter mammalian cells via specific endocytotic pathways, with both lipid-raft and caveolae-mediated pathways being implicated (Fig. 5). Further, the presence of clathrin was found to assist uptake into vertebrate cells (Fig. 6). Clathrin-coated vesicles play a fundamental role in eukaryotic cells. They internalize selected cell-surface molecules by receptor-mediated endocytosis [49]. The clathrin-dependent endocytotic pathway is the best characterised specific endocytotic pathway [50]. Both the immunofluorescence results, and quantification of the amount of Ure2 taken up into cells using its intrinsic enzymatic activity, indicate that the presence of clathrin increases the amount of Ure2 taken up into DKOR cells, particularly for protofibrils. Further, the increased cytoxicity of protofibrils correlates with the increased amount of protofibrils detected within cells. This suggests that protofibrils are endocytosed more efficiently than other aggregate types and/or that they are less prone to degradation during the process of incubation, uptake and accumulation within the cells. This is consistent with the finding that fibril length correlates with the ability to disrupt membranes and to reduce cell viability for a number of amyloidogenic proteins [51]. Interestingly, these results suggest that the uptake mechanism of Ure2 may resemble the internalization mechanism of the mammalian prion protein, PrP, where lipid-raft, calvaeolae and clathrin-mediated mechanisms have all been implicated [52], [53]. These three pathways are not mutually exclusive and may cooperate to uptake the same antigen under different conditions. Here we observe reduced uptake of Ure2 aggregates in the absence of clathrin. This is similar to B cell receptor uptake, which was in addition found to utilize both raft and actin-dependent non-clathrin pathways [54].

In order to understand the mechanism by which the aggregates induce cell damage and death, we examined the effects of aggregates on the cell membrane, and also looked for signs of apoptosis. We found that protofibrils of Ure2 cause cell death by triggering the apoptotic pathway, rather than by causing necrosis, which agrees with observations with Ure2 in murine H-END cells [31] and other aggregates in other cell types [35]–[37]. A growing body of evidence suggests that an increase in membrane permeability induced by amyloid protofibrils may represent a common, primary mechanism of pathogenesis in amyloid-related degenerative diseases [13]. We therefore used patch clamp, which is the classic method to detect changes in the permeability of the cell membrane. Our results indicate that only protofibrils induced an increase in membrane conductivity, with no detectable change in the case of the native dimer or mature fibrils of Ure2 (Fig. 8). In electron microscopy experiments with immuno-labelling of Ure2 aggregates (data not shown), formation of pores within the membrane could not be detected, whereas this has been reported for Aβ, α-synuclein, IAPP, polyglutamine, and PrP [55]–[61]. However, our results are in general agreement with the suggestion that amyloids permeabilize membranes and that the specific structural properties of protofibrils contribute to the efficiency of this activity. Changes in the membrane could in turn induce intracellular signal transduction pathways, leading to the apoptosis response that was detected in protofibril-treated cells (Fig. 10). These early changes at the membrane may initiate a series of downstream pathological events that represent a common pathway for degeneration in amyloid-related diseases. Indeed, consistent with the cytotoxicity and apoptosis results, extracellular addition of protofibrils caused a significant increase in cytosolic free Ca^2+^, whereas equivalent amounts of native dimer had no detectable effect (Fig. 9). The increase in intracellular Ca^2+^ levels may result from an increase in membrane permeability, but it could also result as a consequence of altered intracellular signaling, as an intracellular Ca^2+^ increase itself could induce many signaling transduction pathways.

Elucidation of the uptake mechanism of amyloid aggregates and which pathways are involved in the toxic mechanism has significant implications for therapeutic development. Targeting downstream pathways may not be effective if there are multiple, parallel pathways. If cytotoxic effects are predominantly mediated from within the cell, then blocking the uptake of extracellular aggregates (or aggregation-prone proteins) into cells might be an effective way to prevent cytotoxicity. In this case, focusing on the uptake mechanism would also be an effective strategy, even if multiple downstream pathways are involved. Recent evidence suggests that the mechanism and machinery for prion propagation may be conserved between yeast and higher eukaryotes [62], [63], and the primary mechanism of pathogenesis in prion and amyloid diseases may have important common features, providing hope for development of broadly applicable therapeutics that will be effective against these devastating diseases. This study suggests that the use of well-characterized models, such as the yeast prion protein Ure2, may provide an important tool in achieving this goal.

## Materials and Methods

### Materials

ThT, Tris, nystatin, filipin, nocodazole, cytochalasin D, reduced glutathione, glutathione reductase, NADPH, 1-chloro-2,4-dinitrobenzene, penicillin, streptomycin, poly-L-lysine, tetracycline and Fluo-3-acetoxymethyl ester were from Sigma. Human SH-SY5Y neuroblastoma cells, human HEK-293 embryonic kidney cells (which have certain neuronal properties [64]), MES 23.5 rat dopaminergic neural cells and human HeLa adenocarcinoma cells were from ATCC. Fetal bovine serum (FBS) and chicken serum were from Hyclone. Dulbecco's modified Eagle medium (DMEM) and RPMI 1640 were from Gibco. All other reagents were local products of analytical grade. Millipore water was used throughout.

### Protein production

Ure2 was produced in *Escherichia coli* with a short His tag and purified under native conditions as described previously [23], except that a French press was used in place of sonication to disrupt the cells. Protein was stored at −80°Cand defrosted in a 25°C water bath immediately prior to use. Samples were prepared in 50 mM Tris-HCl buffer containing 0.2 M NaCl and 5% glycerol and centrifuged at 30,000 *g* for 30 min at 4°C to remove any aggregated protein.

### Amyloid formation

The kinetics of amyloid formation of Ure2 proteins was monitored using ThT binding fluorescence as described previously [19], [27]. Incubation was at a constant temperature of 4°C without shaking. Samples were incubated in parallel whenever possible. When comparing the time course of amyloid formation by ThT binding and AFM, samples were taken simultaneously from the same reaction vessel whenever possible. The Ure2 aggregates were also isolated for visualization by atomic force microscopy or electron microscopy. Different Ure2 amyloid species (protofibrils or mature fibrils) were isolated by centrifugation at 18,000 *g* for 30 min at 4°C. Then, the soluble Ure2 concentration in the supernatant fraction was determined by absorbance at 280 nm as described [23]. The concentration of Ure2 fibrillar species was obtained by subtraction of the initial total soluble Ure2 concentration in the original sample and supernatant fraction after formation of fibrillar species.

### Atomic force microscopy

AFM was used to analyze the change in morphology of Ure2 aggregates over time and was carried out essentially as described [27]. A 10 µl drop of the protein sample was deposited on freshly cleaved mica, allowed to stand for 5 min in air, and then washed with two 100 µl aliquots of distilled deionized water, before drying for 5 min in a stream of nitrogen. Tapping mode AFM was performed using a Nanoscope IIIa Multimode-AFM instrument (Digital Instruments) under ambient conditions. Super-sharp silicon tips (Silicon-MDT Ltd) with resonance frequency of about 106 kHz were used at a scan rate of 1–2 Hz. Once the tip was engaged, the set point value was adjusted to minimize the force exerted on the sample while maintaining the sharpness of the image. Samples were imaged at scan sizes between 1 µm and 10 µm using line scan rates below 2 Hz and 512×512 pixels were collected per image.

### Electron microscopy

5-µl aliquots of the fibril suspension were applied to carbon-coated copper grids for 1 min. The grids were blotted, washed twice in droplets of double distilled water, stained with 1% uranyl acetate, and observed with a Phillips Tecnai 20 electron microscope at 120 kV.

### Circular dichroism

Spectra were recorded over the range 200–250 nm in a Pistar-180 Spectrometer (Applied Photophysics, UK). Samples were removed and centrifuged at 30,000 *g* for 30 min to remove large aggregates. Spectra of 20 µM native and protofibrillar Ure2 were measured at 25°C in a 0.1 mm path-length thermostatted cuvette. Data were acquired with a step size of 1 nm. For each sample, the mean residue ellipticity shown is the average of three scans.

### Cell culture

SH-SY5Y, HEK-293 and HeLa cells were cultured in DMEM, supplemented with 10% FBS, 100 U/ml penicillin, and 100 µg/ml streptomycin. MES 23.5 cells were coated with poly-L-lysine (10 µg/ml), and cultured in DMEM supplemented with 20% FBS, 100 U/ml penicillin and 100 µg/ml streptomycin. The cells were incubated at 37°C in humidified 5% CO_2_. The DKOR cell line was maintained in RPMI 1640 with 10% FBS, 1% chicken serum, 10 mM HEPES, and 50 µM 2-mercaptoethanol [33].

### Assay of cellular toxicity

The cells were plated onto 96-well plates and incubated with different states of Ure2 for 48 h. Aggregate cytotoxicity was assessed using the MTT reduction inhibition assay [65]. The absorbance of blue formazan was measured at 590 nm using an automatic plate reader (Thermo). Control experiments were performed by exposing cells to solutions of an equivalent volume of the same buffer for the same length of time. Cell growth curves were obtained by counting the number of cells. The cells were plated onto 24-well plates and incubated with different states of Ure2. The cell number per ml of medium was counted using a hemacytometer and recorded every day until the eighth day.

### Immunofluorescence

SH-SY5Y, HEK-293, HeLa and MES 23.5 cells were grown on glass coverslips in 24-well plates. After 48 h incubation with different states of Ure2, the cells were fixed with 4% paraformaldehyde and 0.1% glutaraldehyde and permeabilized with 0.3% Triton X-100. The cells were then incubated overnight at room temperature with polyclonal anti-rabbit antibody against Ure2 (1∶2000) followed by incubation at room temperature for 2 h with FITC/anti-rabbit IgG (1∶100) and then subjected to confocal microscopy using a Nikon Microphot-FXA confocal laser scanning microscope. Detection of clathrin in DKOR cells was performed as described previously [54]. Images were processed using Adobe Photoshop software.

### Apoptosis assay

Caspase-3 activity was measured using a kit from Clontech. After 24 h incubation with different states of Ure2, SH-SY5Y cells were lysed with a buffer containing 10 mM Tris–HCl, pH 7.4, 10 mM NaCl, 1 mM PMSF, 1 mM EDTA and 0.01% (w/v) SDS. Samples were then centrifuged at 12,000 *g* for 20 min and the protein concentration was determined by the BCA method (Pierce). Aliquots of cytosolic extracts (20 µg protein in 100 µl caspase-3 assay buffer consisting of 50 mM Hepes, pH 7.4, 100 mM NaCl, 0.1% CHAPS, 10 mM DTT, 1 mM EDTA, and 10% glycerol) were mixed with equal volumes of 40 µM colorimetric tetrapeptide substrate (Ac-DEVD-pNA) in the same buffer and monitored using an ELISA plate reader (Thermo). To detect early sign of apoptosis, an annexin V and PI double-staining kit was used (Keygen biotech). Cells were washed with phosphate-buffered saline once and then stained with annexin V and 1 µg/ml PI for 10 min at room temperature and then subjected to fluorescence microscopy.

### Endocytosis inhibition assay

MES 23.5 cells were grown on glass coverslips in 35 mm culture dishes (Nunc). 3 µM Ure2 protofibrils and 100 µg/ml nystatin, 5 µg/ml filipin, 50 µmol/l nocodazole or 50 µg/ml cytochalasin D were added extracellularly. After 48 h of incubation, the cells were subjected to immunofluorescence assay as described above.

### Patch clamp

Silver chloride wires were used as electrodes to apply voltages and record currents across the cell membrane. The rear-chamber potential was taken as ground, and the additions were made to the front chamber. For measurements of membrane conductance a ramp protocol (−100 to +100 mV, 50 mV/s) was used. Voltages were generated and currents digitized at a resolution of 12 bits by an AD Laboratory ADC/DAC board running software written in the laboratory. Currents were transduced by an Axopatch 200 A amplifier (Axon Instruments, Foster City, CA) connected to the AD Laboratory board. Buffer, native Ure2, mature fibrils and protofibrils were added sequentially to SH-SY5Y cells. Increasing concentrations of protofibrils were added to record the *I-V* curve.

### Intracellular free Ca^2+^ concentration

Prior to Ure2 addition, SH-SY5Y cells were incubated at 37°C for 5 min with 5.0 µM Fluo-3-acetoxymethyl ester which labels free Ca^2+^. The cells then were washed with serum-free DMEM without phenol red to remove the excess probe and mounted on the stage of the confocal microscope. Native Ure2, protofibrils or mature fibrils of Ure2 were added to the cells, and Fluo-3 fluorescence was monitored at 488 nm 5 min after Ure2 addition using the software Time Course Kinetic (Olympus). The same field was imaged from 30 min to 4 h. Fluorescence quantification was performed using Quantity One software (Bio-Rad).

### Quantification of Ure2 uptake

Clathrin-expression in DKOR cells was inhibited by incubation of the cells with 100 ng/ml doxycycline for 72 h prior to assay [33]. Clathrin-expressing and clathrin-depleted cells were incubated with different states of Ure2 for 48 h and then lysed as described above (see Apoptosis assay). The quantity of different states of Ure2 entering into cells was determined by assay of glutaredoxin enzymatic activity, as described previously [22]. The reaction conditions were 1.5 µM Ure2, 1.5 µM aggregates or 100 µl cell lysate, 100 mM sodium phosphate, pH 7.5, 1.5 mM reduced glutathione, 3 mM 2-hydroxyethyl disulfide, 0.5 units/ml glutathione reductase, 0.2 mM NADPH, and 1 mM EDTA at 25°C. Lysates of clathrin-expressing and clathrin-depleted cells which were not incubated with Ure2 or aggregates were also assayed as controls. The glutaredoxin activity of Ure2 or its aggregates in the cell lysate was obtained by subtracting the activity of the respective control.
